# Protein Engineering of Dual-Cys Cyanobacteriochrome AM1_1186g2 for Biliverdin Incorporation and Far-Red/Blue Reversible Photoconversion

**DOI:** 10.3390/ijms20122935

**Published:** 2019-06-15

**Authors:** Yuto Kuwasaki, Keita Miyake, Keiji Fushimi, Yuka Takeda, Yoshibumi Ueda, Takahiro Nakajima, Masahiko Ikeuchi, Moritoshi Sato, Rei Narikawa

**Affiliations:** 1 Graduate School of Arts and Sciences, University of Tokyo, Komaba, Meguro, Tokyo 153-8902, Japan; kuwasakiyuto@g.ecc.u-tokyo.ac.jp (Y.K.); yoshibumiueda@gmail.com (Y.U.); ctnaka@mail.ecc.u-tokyo.ac.jp (T.N.); mikeuchi@bio.c.u-tokyo.ac.jp (M.I.); cmsato@mail.ecc.u-tokyo.ac.jp (M.S.); 2 Graduate School of Science and Technology, Shizuoka University, Ohya, Suruga-ku, Shizuoka 422-8529, Japan; miyake.keita.17@shizuoka.ac.jp (K.M.); fushimi.keiji@shizuoka.ac.jp (K.F.); takeda.yuka.14@shizuoka.ac.jp (Y.T.); 3 Core Research for Evolutional Science and Technology, Japan Science and Technology Agency, 4-1-8 Honcho Kawaguchi, Saitama 332-0012, Japan; 4 Japan Agency for Medical Research and Development-PRIME, Tokyo 100-0004, Japan; 5 Green Biology Research Division, Research Institute of Green Science and Technology, Shizuoka University, Ohya, Suruga-ku, Shizuoka 422-8529, Japan

**Keywords:** bilin, site-directed mutagenesis, reversible Cys adduct formation

## Abstract

Cyanobacteria have cyanobacteriochromes (CBCRs), which are photoreceptors that bind to a linear tetrapyrrole chromophore and sense UV-to-visible light. A recent study revealed that the dual-Cys CBCR AM1_1186g2 covalently attaches to phycocyanobilin and exhibits unique photoconversion between a Pr form (red-absorbing dark state, λ_max_ = 641 nm) and Pb form (blue-absorbing photoproduct, λ_max_ = 416 nm). This wavelength separation is larger than those of the other CBCRs, which is advantageous for optical tools. Nowadays, bioimaging and optogenetics technologies are powerful tools for biological research. In particular, the utilization of far-red and near-infrared light sources is required for noninvasive applications to mammals because of their high potential to penetrate into deep tissues. Biliverdin (BV) is an intrinsic chromophore and absorbs the longest wavelength among natural linear tetrapyrrole chromophores. Although the BV-binding photoreceptors are promising platforms for developing optical tools, AM1_1186g2 cannot efficiently attach BV. Herein, by rationally introducing several replacements, we developed a BV-binding AM1_1186g2 variant, KCAP_QV, that exhibited reversible photoconversion between a Pfr form (far-red-absorbing dark state, λ_max_ = 691 nm) and Pb form (λ_max_ = 398 nm). This wavelength separation reached 293 nm, which is the largest among the known phytochrome and CBCR photoreceptors. In conclusion, the KCAP_QV molecule developed in this study can offer an alternative platform for the development of unique optical tools.

## 1. Introduction

Cyanobacteria, prokaryotes that perform oxygenic photosynthesis, have various photoreceptors to detect and acclimate to ambient light conditions. In cyanobacteria, linear tetrapyrrole-binding photoreceptors, phytochromes, and cyanobacteriochromes (CBCRs), play central roles in these acclimation processes [1,2,3,4,5,6,7,8,9,10]. Phytochromes are widespread in a broad range of organisms, including plants, algae, cyanobacteria, fungi, and non-photosynthetic bacteria [11,12]. They have a conserved cGMP-phosphodiesterase/adenylate cyclase/FhlA (GAF) domain to attach to linear tetrapyrrole chromophores and detect red and far-red light in most cases. The photosensory core module of most phytochromes comprises three domains: Per/Arnt/Sim (PAS), GAF, and phytochrome-specific (PHY) domains. Most cyanobacterial phytochromes, such as Cph1, also have these three domains, but some unusual phytochromes without the N-terminal PAS domain have been detected in cyanobacteria.

The cyanobacteriochromes (CBCRs) are phytochrome-like photoreceptors that have to date been detected only from cyanobacteria; they are involved in various light acclimation processes, such as phototactic motility, chromatic acclimation, and light-dependent cell aggregation [1,2,3,4,5,8,9,10,13]. In contrast to the phytochromes, only the GAF domain is needed for chromophore incorporation and proper photoconversion [12,13,14]. Most CBCRs have a canonical Cys residue to bind a linear tetrapyrrole chromophore irreversibly and show reversible photoconversion that is triggered by *Z*/*E* isomerization of the C15-C16 double bond between the C and D rings of the chromophore. Various photoconvertible CBCRs covering the ultraviolet-to-far-red region of the spectrum have been identified [15,16,17,18,19,20,21,22,23,24,25,26,27]. These CBCRs are categorized into certain lineages based on their primary sequences.

All CBCR GAF domains except those belonging to the Asp-Xaa-Cys-Ile-Pro (DXCIP) lineage possess a canonical Cys residue that is stably ligated to ring A of the chromophore [12,27]. Furthermore, several CBCR GAF domains have a conserved second Cys residue, which is transiently ligated to C10 of the chromophore [16,28,29,30,31]. Ligation to C10 results in a pronounced shortening of the π-electron conjugated system and the absorption of the blue light region. Several dual-Cys CBCRs have been identified from several lineages [16,23,24,26]. Among them, Asp-Xaa-Cys-Phe (DXCF) and insert-Cys CBCRs are representative ones showing such reversible Cys ligation [16,23,31]. Typical DXCF CBCRs have a second Cys residue within the highly conserved DXCF motif and show reversible photoconversion between a blue-absorbing 15*Z* dark state (Pb) and a green-absorbing 15*E* photoproduct (Pg) [28,29,30]. The second Cys residue is ligated to the chromophore in the dark state and free from the chromophore in the photoproduct [28,29,30]. Sensing light qualities of the photoproducts are highly diversified, covering the teal-to-orange region depending on their binding chromophore species and specific color-tuning mechanisms [20,21,22,32,33,34]. Conversely, insert-Cys CBCRs have a second Cys residue within a large loop insertion and show reversible photoconversion covering the UV-to-orange region [23,35].

**Figure 1:**
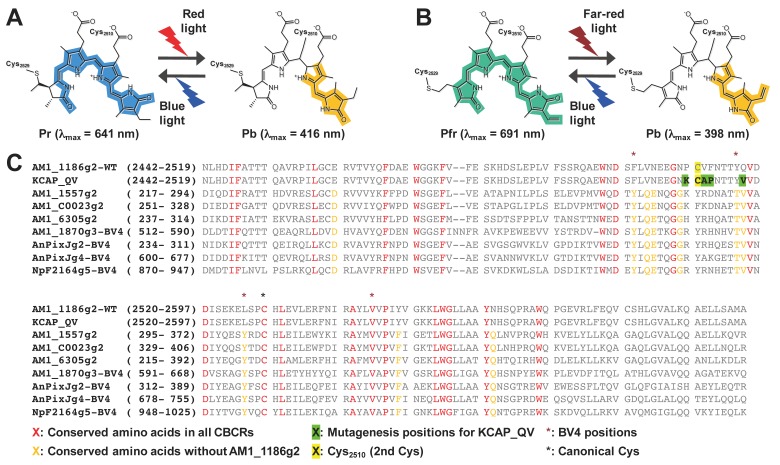
Photocycle models and multiple alignment. (**A**) Photocycle model of the PCB-binding AM1_1186g2 (**B**) Photocycle model of the BV-binding AM1_1186g2 KCAP_QV (Pro_2509_Lys, Val_2511_Ala, Phe_2512_Pro, and Gln_2517_Val) variant. Color highlights represent π-conjugated system. (**C**) Multiple alignment of AM1_1186g2 wild-type (WT) and KCAP_QV with the other BV-binding CBCRs.

We recently reported another example, AM1_1186g2, showing reversible Cys ligation during photoconversion. AM1_1186g2 is categorized into the expanded red/green (XRG) lineage, covalently binds phycocyanobilin (PCB), and shows red/blue reversible photoconversion (Figure 1A) [24]. Typical XRG CBCRs that have no second Cys, such as AnPixJg2, bind PCB and photoconvert between a red-absorbing 15*Z* dark state (Pr) and a green-absorbing 15*E* photoproduct (Pg) [17]. AM1_1186g2, however, possesses a second Cys residue within the α3-helix, which is a unique position, and photoconverts reversibly between a Cys-free red-absorbing 15*Z* dark state (Pr, λ_max_ = 641 nm) and a Cys-adducted blue-absorbing 15*E* photoproduct (Pb, λ_max_ = 416 nm). The wavelength separation is the largest among the known CBCRs.

Some typical XRG CBCRs detected from the cyanobacterium, *Acaryochloris marina*, covalently bind not only PCB but also biliverdin (BV), which is a precursor of PCB in the biosynthetic pathway from heme [36,37,38]. Since BV has a longer π-electron conjugated system than PCB, BV-binding ones show reversible photoconversion between a far-red-absorbing 15*Z* dark state (Pfr) and an orange-absorbing 15*E* photoproduct (Po). Based on a sequence comparison between the BV-binding and non-BV-binding ones, we predicted residues involved in BV incorporation. Site-directed mutagenesis and structural analyses elucidated that a cooperative rearrangement of four replaced residues (BV4) well escapes steric hindrance to ring C [38]. Although we succeeded in BV incorporation of AnPixJg2, AnPixJg4, AM1_1870g3, and NpF2164g5 variants by introducing these BV4 residues, we failed to obtain a BV-binding AM1_1186g2 variant in a previous study.

BV-binding photoreceptors are advantageous for optogenetic control in mammals because BV is an endogenous molecule in mammals and absorbs far-red light, which penetrates into the deep tissues of mammals. The large separation of the two absorbing forms of AM1_1186g2 enables us to achieve full photoconversion, which is favorable for strict optogenetic control. In the present study, we obtained an AM1_1186g2 variant called AM1_1186g2_KCAP-QV that efficiently incorporated BV and showed far-red/blue reversible photoconversion (Figure 1B).

## 2. Results

### 2.1. RCAP: Efficient BV Incorporation

The AM1_1186g2 wild-type (WT) covalently binds PCB and exhibits unique photoconversion between a Pr dark state and a Pb photoproduct (Figure 1A) [24]. Irradiation of the Pr form with red light results in photoisomerization of the C15-16 double bond of PCB, and transient formation of an Io form, which is intermediate immediately after the photoisomerization. Thereafter, the second Cys residue (Cys_2510_) slowly forms a thioether linkage to the C10 of PCB to generate the blue-shifted Pb form. Although the WT protein accumulated the Io form during Pr-to-Pb photoconversion, the PR protein in which the Pro_2509_ was replaced with Arg showed quick Pr-to-Pb photoconversion without noticeable accumulation of the Io form [24]. Since the Pro_2509_ is positioned just before the second Cys residue (Figure 1C), this replacement may largely affect the positioning of the second Cys residue, enabling rapid covalent bond formation.

**Figure 2:**
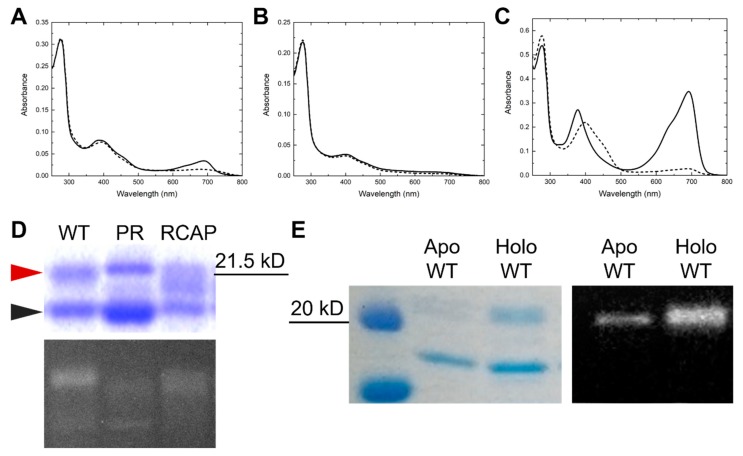
Acquisition of the RCAP variant protein (**A**–**C**). Absorption spectra of the BV-binding AM1_1186g2 wild-type ((**A**), WT), P_2509_R ((**B**), PR), and RCAP variants ((**C**), RCAP). The solid and dashed lines correspond to the dark states and the photoproducts responding to the far-red light, respectively. (**D**) Coomassie Brilliant Blue (CBB)-stained gel and in-gel Zn-dependent fluorescence assay of WT, PR, and RCAP. Red and black arrowheads indicate holoproteins of BV-binding AM1_1186g2 variants and contamination proteins, respectively. (**E**) CBB-stained gel (left) and Western blotting analysis using antibodies against His-tag (right) of AM1_1186g2 apoprotein and holoprotein.

To evaluate the BV-binding capability of the WT protein, we previously expressed the WT protein in the BV-producing *Escherichia coli* and found that the WT protein could slightly bind BV and absorb light in the far-red region (Figure 2A) [38]. Notably, irradiation with far-red light resulted in the bleaching of the far-red light absorption, but not an increase in the blue light region, suggesting no covalent bond formation between the second Cys residue and the C10 of the chromophore, in contrast to the PCB-binding one (Figure 2A). The BV-binding pocket may be arranged in comparison with the case for PCB incorporation, which may result in the inefficient BV incorporation and inaccessibility of the second Cys residue to the C10. Since the Pro_2509_Arg replacement improved the accessibility of the second Cys residue to the C10 of the PCB, we expected similar improvement for the BV incorporation. The PR replacement, however, resulted in almost no BV binding (Figure 2B).

Therefore, we considered that the PR replacement alone is not enough for the BV incorporation and covalent bond formation. In this context, we next focused on two residues just after the second Cys residue: Val_2511_ and Phe_2512_. Since covalent bond formation of the second Cys residue, Cys_2510_, with the C10 may cause steric hindrance between the next Val_2511_ residue and the ring C carbonyl, Ala possessing a smaller side chain may be favorable for BV incorporation. Further, for structural modification around the second Cys residue, we replaced Phe_2512_ with Pro, which has a unique ring structure, and thus is likely to promote structural modification. The obtained mutant named RCAP possessing three substitutions (Pro_2509_Arg, Val_2511_Ala, and Phe_2512_Pro) showed moderately high BV-binding capability (Figure 2C). The BV-binding RCAP protein exhibited reversible photoconversion between a far-red-absorbing form (Pfr, λ_max_ = 691 nm) and a blue-light-absorbing form (Pb, λ_max_ = 398 nm; Figure 2C). The Pfr form is 50 nm red-shifted compared with the Pr form binding PCB, which is consistent with the absorption of BV and PCB themselves. Conversely, the Pb form of the BV-binding RCAP was blue-shifted compared with that of the PCB-binding one. This blue shift might be derived from overlap between the Soret band and blue absorption. Next, to address the purity and BV-binding efficiency of the AM1_1186g2 variant proteins, these proteins were subjected to SDS-PAGE (Figure 2D). There were two main protein bands around 20 kDa based on the Coomassie Brilliant Blue (CBB) staining. The protein bands just below 21.5 kDa (red arrowhead) emitted fluorescence in the presence of zinc ion, indicating that these protein bands corresponded to the BV-binding holoproteins. To confirm that the bands below the holoprotein bands (black arrowhead) corresponded to the apoproteins, we purified the WT proteins from both non-BV-producing and BV-producing *E. coli* for His-tag immunodetection. The bands around 20 kDa did not react with the His-tag antibody, and so were concluded not to be derived from the apoproteins but from the contaminant nonspecific proteins (Figure 2E). Since the holoprotein bands of the RCAP protein were not single but smeared in comparison with the WT and PR proteins (Figure 2D), the RCAP holoprotein should be unstable and easily degraded.

### 2.2. KCAP: Improvement in Protein Stability

**Figure 3:**
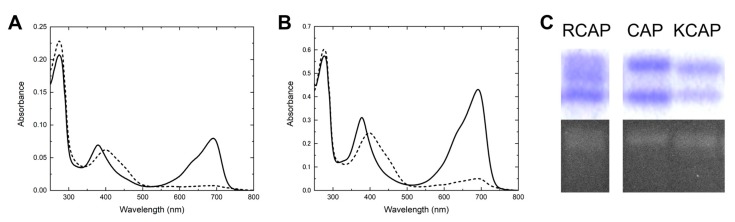
Acquisition of the KCAP variant protein. (**A**,**B**) The absorption spectra of the AM1_1186g2-CAP (**A**) and KCAP variants (**B**). The solid and dashed lines correspond to the dark states and the photoproducts responding to the far-red light, respectively. (**C**) Coomassie Brilliant Blue (CBB)-stained gel (upper) and in-gel Zn-dependent fluorescence assay (lower) of the RCAP (Pro_2509_Arg, Val_2511_Ala, and Phe_2512_Pro), CAP, and KCAP proteins.

Judging from the absorption spectra and zinc-induced fluorescence of the WT and PR proteins (Figure 2A,B,D), Pro_2509_Arg replacement based on the WT background had an inhibitory effect on the BV-binding efficiency and/or purity. In this context, Arg_2509_ of the RCAP protein may have a similar inhibitory effect. To verify this possibility, we focused on this residue for further improvement. We replaced the Arg residue with the original Pro or Lys residue, which has a positive charge like Arg, producing CAP (Val_2511_Ala, and Phe_2512_Pro) and KCAP (Pro_2509_Lys, Val_2511_Ala, and Phe_2512_Pro) variant proteins. CAP and KCAP variant proteins showed reversible far-red/blue photoconversion, like the RCAP protein (Figure 3A,B). The specific absorbance ratio [SAR, (peak red-band absorption)/(peak 280 nm absorption)] value of the CAP protein was lower than that of the RCAP protein (Table 1), suggesting that the Pro_2509_Arg replacement based on the CAP background did not have inhibitory effects on the BV-binding efficiency and/or purity, in contrast to the case for the WT protein. Conversely, the SAR value of the KCAP was higher than that of the RCAP protein (Table 1). Furthermore, judging from the CBB-stained and zinc-induced fluorescent gels (Figure 3C), the purified CAP and KCAP proteins were found to be present not as smears but as single bands, indicating that these variant holoproteins are stable in comparison with the RCAP protein. In conclusion, the KCAP protein is the best variant showing high BV-binding efficiency and holoprotein stability at this time.

### 2.3. KCAP_QV: Improvement in Holoprotein Expression

**Figure 4:**
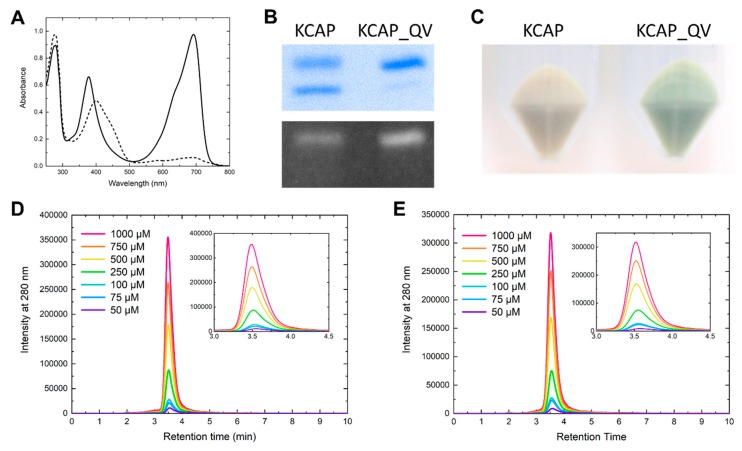
Acquisition and characterization of the KCAP_QV variant protein. (**A**) The absorption spectrum of the AM1_1186g2-KCAP_QV variant. The solid and dashed lines correspond to the dark state and the photoproduct responding to the far-red light, respectively. (**B**) Coomassie Brilliant Blue (CBB)-stained gel (upper) and in-gel Zn-dependent fluorescence assay (lower) of the KCAP and KCAP_QV proteins. (**C**) *E. coli* cell pellets expressing His-tagged KCAP and KCAP_QV proteins. (**D**,**E**) HPLC analyses by gel filtration chromatography for estimating the oligomeric state of KCAP_QV of the Pfr (**D**) and Pb (**E**) forms. The native proteins adjusted to 50 (purple), 75 (blue), 100 (blue green), 250 (green), 500 (yellow), 750 (orange) and 1000 µM (magenta) were injected as both the Pfr and Pb forms. These chromatograms were recorded at 280 nm. The molecular sizes were calculated by standard curve constructed from retention times of marker proteins. Insets of (**D**,**E**) are focused on the peak area.

As the next approach for improving BV-binding capability, we compared the primary sequence of the KCAP protein with those of other BV-binding CBCRs (Figure 1C). We previously found and developed seven BV-binding CBCRs [36,37,38]. We hypothesized that some amino acid residues conserved in these CBCRs but not in the KCAP protein might be crucial for BV-binding capability. Based on multiple sequence alignment and structural information, we identified seven amino acid residues (Phe_2501_, Val_2503_, Asn_2504_, Asn_2508_, Tyr_2516_, Gln_2517_, and Leu_2526_) near the chromophore that are unique to the KCAP protein. Among these residues, Phe_2501_, Tyr_2516_, and Leu_2526_ correspond to the BV4 residues. Since the introduction of the BV4 residues into the WT protein failed to achieve any improvement in BV-binding capability [38], we excluded these three residues from further targeting. Thus, we replaced the other four residues with residues conserved among the other BV-binding CBCRs one by one: KCAP_VQ (Val_2503_Gln, Pro_2509_Lys, Val_2511_Ala, and Phe_2512_Pro), KCAP_NE (Asn_2504_Glu, Pro_2509_Lys, Val_2511_Ala, and Phe_2512_Pro), KCAP_NG (Asn_2508_Gly, Pro_2509_Lys, Val_2511_Ala, and Phe_2512_Pro), and KCAP_QV (Pro_2509_Lys, Val_2511_Ala, Phe_2512_Pro, and Gln_2517_Val). Among them, only one mutant, KCAP_QV, had a higher SAR value (1.09) than the background KCAP protein (Figure 4A), whereas the other three variant proteins had lower ones (Figure S1). In particular, Asn_2504_Glu replacement (KCAP_NE) resulted in the loss of photoconversion capability and protein aggregation induced by far-red light illumination (Figure S1B). Judging from the SAR values, the KCAP_QV protein is the best variant for BV incorporation among the variants tested in this study.

To clarify the details of the improvement by Gln_2517_Val replacement, the KCAP and KCAP_QV proteins were subjected to SDS-PAGE. The main contaminant protein band that appeared for the KCAP preparation was almost undetectable for the KCAP_QV preparation (Figure 4B), indicating that the higher SAR value is mainly derived from higher yield of the holoprotein expression and resultant improvement of purity. To verify this assumption, we compared the *E. coli* cell pellets expressing the KCAP or KCAP_QV protein. As a result, the cell pellet expressing the KCAP_QV protein showed a deep green color, whereas that expressing the KCAP protein showed a pale green color (Figure 4C). From these results, we concluded that Gln_2517_Val mutation markedly improved the expression yield of the BV-binding holoproteins. Since high purity of the KCAP_QV protein enables precise determination of the BV-binding efficiency, we calculated the BV-binding efficiency as 67% by the method described in a previous study [38]. Since this value is comparable to those of the other BV-binding molecules developed in the previous study [38], we stopped our engineering efforts and regarded the KCAP_QV protein as the final developmental product in this study.

To characterize the photochemical properties of the KCAP_QV protein, acid-denatured spectra were measured. Its Pfr form denatured by guanidinium chloride under acidic conditions exhibited an absorption peak at 708 nm, and white light illumination did not result in any spectral change (Figure S2A). This result showed that the Pfr form attached to the 15*Z*-isomer of BV. On the other hand, that of the Pb form showed an absorption peak at 637 nm, and white light illumination resulted in a red shift peaking at 708 nm (Figure S2B). This showed that the Pb form bound the 15*E*-isomer of BV.

Furthermore, we measured the photoconversion kinetics of both direction and reversibility with repetitive conversions. The absorbance change at 691 nm was monitored at room temperature during repetitive far-red and blue light illuminations (Figure S3). The Pfr-to-Pb photoconversion kinetics was extremely slow in comparison with the opposite Pb-to-Pfr kinetics. Further, the Pfr-to-Pb kinetics was found to be very slow when compared also with the Pfr-to-Po kinetics of AnPixJg2-BV4 (Figure S4). This slow Pfr-to-Pb photoconversion kinetics was conserved among the BV-binding AM1_1186g2 variant proteins produced in this study (data not shown). Unlike the PCB-binding AM1_1186g2 wild-type protein, we could not detect any intermediate form during the Pfr-to-Pb conversion (data not shown), indicating that the quantum yield of the *Z*/*E* isomerization may be quite low. Repetitive photoconversion resulted in a decrease in the Pfr peak absorbance (Figure S3), indicating that prolonged irradiation with far-red light causes bleaching of the KCAP_QV protein. Although we incubated the Pb photoproduct under dark conditions at room temperature for 9 h, we could not detect obvious dark reversion to the Pfr dark state (data not shown).

To elucidate oligomeric states of the both forms of the KCAP_QV protein, we performed HPLC analysis using a gel filtration column. The peaks of all preparations were detected around 3.5 min of the retention time. The retention times of the both forms became a little larger as higher concentration (Figure 4D,E). Molecular sizes relative to the theoretical monomeric form (22.4 kDa) was ranging from 1.1 to 1.3 (24–29.5 kDa) for the Pfr form and from 1.0 to 1.2 (21.5–26.5 kDa) for the Pb form. Although these data might mean that only a small population of the proteins formed a dimer at higher concentration, the majority of the proteins in both forms were present as a monomer. Slight differences in retention times of the two forms even at the same concentration might reflect some structural change depending on the photoconversion.

## 3. Discussion

In this work, we engineered dual-Cys CBCR AM1_1186g2 to accept the mammalian intrinsic chromophore biliverdin by replacing several amino acids. The engineered molecule, KCAP_QV, efficiently incorporated the BV with high holoprotein stability and expression yield. The KCAP_QV showed reversible photoconversion between the Pfr dark state and the Pb photoproduct. The wavelength separation of the two forms was 293 nm, which is the largest among the known phytochromes and CBCRs.

We previously succeeded in identifying four residues (BV4) crucial for BV incorporation of some of the XRG CBCRs [38]. Although AM1_1186g2 also belongs to the XRG lineage, the same BV4 replacement on AM1_1186g2 failed to improve BV incorporation [38]. Since AM1_1186g2 showed reversible covalent bond formation between the second Cys residue and the C10 of the chromophore, which is a unique feature of AM1_1186g2 among the XRG CBCRs, the structural arrangement near the chromophore should be quite different from those of the canonical XRG CBCRs, probably leading to the failure.

In the case of the BV4 variant proteins, the canonical Cys residue stably ligates to the C3^2^ of the BV chromophore, whereas the same residue ligates to the C3^1^ of the PCB chromophore [28,38]. This unique binding mode resulted in deeper BV insertion into the protein pocket. Although we could not yet reveal the structures of the PCB- and BV-binding AM1_1186g2 variant proteins, a similar situation may be expected in the case of AM1_1186g2. In this context, the mutated residues in this study may contribute to escaping the steric hindrance derived from the deeper BV insertion into the protein pocket and thus improving the BV-binding efficiency. The reversible covalent bonding site, C10, is present between the rings B and C possessing bulky propionate side chains. The replacement of residues near the second Cys residue (Pro_2509_Lys, Val_2511_Ala, and Phe_2512_Pro) may be crucial for escaping the steric hindrance with these propionate side chains. Notably, Pro_2509_Lys replacement resulted in an improvement of holoprotein stability (Figure 3C), although replacement with a similar Arg residue conversely led to holoprotein instability. At present, it is difficult to provide a plausible interpretation of this without some structural information.

We further identified that the Gln_2517_Val replacement based on the KCAP background made a major contribution to holoprotein expression yield, resulting in the highest purity and SAR value among the variant proteins analyzed in this study (Figure 4). Gln_2517_Val position corresponds to the residue just after one of the BV4 positions, Phe_308_Thr, based on multiple alignment and structural information. Thus, this replacement may modify the binding pocket near ring C to facilitate holoprotein expression.

Thus, the KCAP_QV variant protein is the best variant based on the AM1_1186g2 protein. Among the known BV-binding CBCR proteins, the KCAP_QV protein possesses unique features not only to show extremely large wavelength separation but also to show very slow photoconversion from the dark state to the photoproduct and not to show dark reversion from the photoproduct to the dark state. The PCB-binding WT protein showed slow photoconversion from the dark state to the photoproduct to accumulate the intermediate Io form. In contrast, the BV-binding KCAP_QV protein showed slow photoconversion without noticeable accumulation of any intermediate, suggesting low quantum yield of the light-induced *Z*/*E* isomerization. Although it is often the case that low quantum yield of the isomerization may result in bright fluorescence, fluorescence quantum yield of the Pfr form was measured to be about 0.7%, as low as those of the other photoconvertible BV-binding CBCRs [37]. Additionally, prolonged irradiation with far-red light resulted in photobleaching of the Pfr form. In this context, improvement in the Pfr-to-Pb photoconversion kinetics is needed for developing stable reversible optical tools. Nonetheless, the BV-binding KCAP_QV protein with unique features should be an alternative platform for the development of BV-based optical tools such as photoacoustic imaging [39] and photochromic FRET [40].

## 4. Materials and Methods

### 4.1. Plasmid Construction

The *Escherichia coli* strain JM109 or Mach1 was used for cloning plasmid DNA. Plasmids expressing His-tagged AM1_1186g2 wild-type and P_2509_R mutant were constructed in a previous study [24]. To perform site-directed mutagenesis, PrimeSTAR MAX mutagenesis kit reagents (TaKaRa, Shiga, Japan) were used with appropriate primers (Table S1) and all AM1_1186g2 variant constructs were obtained. The sequences of all constructs were confirmed by DNA sequencing.

### 4.2. Expression and Purification of His-Tagged AM1_1186g2 Variants

*E. coli* strain C41 (Novagen, Madison, WI, USA) harboring the pKT270 plasmid to produce BV was used for the expression of BV-binding AM1_1186g2 variants. For the expression of AM1_1186g2 apoprotein, we used the C41 strain without the pKT270 plasmid. As a pre-culture, one colony was picked up and incubated at 37 °C overnight in 10 mL of LB medium with 20 μg/mL kanamycin and/or 20 μg/mL chloramphenicol. Each culture was added into 1 L of LB medium with 20 μg/mL kanamycin and/or 20 μg/mL chloramphenicol and incubated until OD_600_ was between 0.4 and 0.8. Subsequently, isopropyl thio-β-d-galactopyranoside was added to a final concentration of 0.1 mM and cultured at 18 ºC overnight for the induction of protein expression. Next, each sample was harvested by centrifugation at 4 °C and frozen at −80 °C for 30 min. Each frozen cell pellet was thawed at 4 °C and suspended in 40 mL of A buffer (20 mM HEPES-NaOH (pH 7.5), 100 mM NaCl, 10% (*w*/*v*) glycerol, and 0.5 mM Tris-carboxyethylphosphine (Pierce)) and disrupted by three passages through an Emulsiflex C5 high-pressure homogenizer at 12,000 psi (Avestin Inc., Ottawa, ON, Canada, Canada). The solution was centrifuged at 165,000 *g* for 30 min at 4 °C, and the supernatant containing His-tagged protein was filtered by 0.8 μm Cellulose Acetate Membranes. The filtrate was passed through a nickel affinity His-trap chelating column (GE Healthcare, Piscataway, NJ, USA). After washing with the A buffer containing 30, 50, and 100 mM imidazole, the His-tagged protein was eluted using a linear imidazole gradient from 100 to 400 mM. Then, the protein solution was incubated for 1 h with 1 mM EDTA. After that, to remove imidazole and EDTA, the protein solution was dialyzed against the A buffer including 1 mM dithiothreitol.

### 4.3. Sodium Dodecyl Sulfate-Polyacrylamide Gel Electrophoresis (SDS-PAGE) and Zinc-Dependent Fluorescence Gel Assay

Purified AM1_1186g2 variants were dissolved in 2% (*w*/*v*) SDS, 60 mM DTT, and 60 mM Tris-HCl, pH 8.0, for SDS-PAGE (12% (*w*/*v*) acrylamide). After denaturation at 95 °C for 3 min, the samples were electrophoresed at room temperature. The gels were stained with CBB R-250.

For the zinc-dependent fluorescence assay, the gels were immersed in distilled water for 30 min, followed by immersion with 20 μM zinc acetate at room temperature for 30 min. Thereafter, zinc-dependent fluorescence was detected using WSE-6100 LuminoGraph (ATTO, Tokyo, JAPAN) through a 600-nm long path filter upon excitation with blue (λ_max_ = 470 nm) and green light (λ_max_ = 527 nm) through a 562-nm short path filter.

### 4.4. Western Blotting Assay

AM1_1186g2 apoprotein and holoprotein were incubated with the SDS-PAGE sample buffer at 95 °C for 5 min; then, samples were loaded in the wells of SDS-PAGE (10% (*w*/*v*) acrylamide) gels. The samples were run at 30 mA for 1 h. The gels were transferred to PVDF membranes with 18 V for 1 h. After incubation with blocking buffer (Blocking One; Nacalai Tesque, Kyoto, Japan) for 1 h at room temperature, anti-His-tag monoclonal antibody (D291-3S; Medical & Biological Laboratories Co., Ltd., Nagoya, Japan) (1:2000) was applied overnight at 4 °C. Next, horseradish peroxidase (HRP)-conjugated Goat Anti-Mouse IgG antibody (ab6789; Abcam, Cambridge, UK) was applied (1:4000) for 1 h. HRP chemiluminescence signals were detected by LAS-3000 mini (Fujifilm Corporation, Tokyo, Japan).

### 4.5. Spectrometry and Measurement of Photoconversion Kinetics

Absorption spectra of all AM1_1186g2 variants were recorded using a model UV-2600 spectrophotometer (Shimadzu, Kyoto, Japan) at room temperature. Far-red and blue monochromatic light was provided using Opt-Spectrum Generator (OSG; Hamamatsu Photonics, Hamamatsu, Japan).

For the acid denaturation of KCAP_QV, 200 μL was diluted into 1 mL of 7 M guanidinium chloride/1% HCl (*v*/*v*), and absorption spectra were recorded before and after 1 min of illumination with white light.

Pfr-to-Pb or Pfr-to-Po photoconversion kinetics of KCAP_QV and AnPixJg2-BV4 were measured by monitoring at 691 nm. Before the measurement, KCAP_QV and AnPixJg2-BV4 were irradiated with light at 430 and 593 nm, respectively, to form a 15*Z* dark state. Ten seconds after the start of measurement, KCAP_QV and AnPixJg2-BV4 were irradiated with far-red light at 691 nm while measuring absorption. To measure reversible photoconversion kinetics of KCAP_QV, the absorbance at 691 nm was monitored during far-red and blue light illuminations. Before the measurement, KCAP_QV was irradiated with blue light at 398 nm to form a 15*Z* dark state. Ten seconds after starting the measurement, KCAP_QV was irradiated with far-red light at 691 nm while measuring absorption. After the Pfr-to-Pb photoconversion, KCAP_QV was irradiated with the blue light at 398 nm for 3 min and then irradiated with far-red light again. This light illumination cycle was repeated three times.

### 4.6. HPLC Analysis

After filtration removing insoluble aggregates, concentrations of KCAP_QV were adjusted to 50, 75, 100, 250, 500, 750 and 1000 μM, respectively. These samples were irradiated with blue (398 nm) and far-red (691 nm) lights to generate Pfr and Pb forms, respectively.

To calculate the molecular sizes of the native protein of each form, gel filtration chromatography was performed using a Prominence HPLC system (Shimadzu, Kyoto, Japan). Each sample was injected in a volume of 10 μL and eluted by using a HPLC column (Inertsil WP300 Diol, 4.6 i.d. × 250 mm, 5 μm; GL Sciences) at 28 °C with a buffer (50 mM phosphate (pH 7.0), 300 mM NaCl), in which these chromatograms were recorded at 280 nm. The molecular sizes were calculated by standard curve constructed from retention times of marker proteins (cytochrome C, 12.4 kD; ovalbumin, 44.3 kD; alcohol dehydrase, 150 kD; β-amylase, 200 kD; blue dextran, 2000 kD).

## Figures and Tables

**Table 1:** Properties of the BV-binding AM1_1186g2 variants. SAR: specific absorption ratio (ABS_691_/ABS_280_).

Name	Dark State (λ_max_)	Photoproduct (λ_max_)	SAR
**WT**	687 nm	398 nm	0.11
**PR**	-	-	0.03
**RCAP**	691 nm	398 nm	0.65
**CAP**	691 nm	398 nm	0.40
**KCAP**	691 nm	398 nm	0.76
**KCAP_V_2503_Q**	690 nm	398 nm	0.61
**KCAP_N_2504_E**	691 nm	398 nm	0.18
**KCAP_N_2508_G**	691 nm	398 nm	0.48
**KCAP_Q_2517_V**	691 nm	398 nm	1.09

## Supplementary Materials

Supplementary materials can be found at https://www.mdpi.com/1422-0067/20/12/2935/s1.
