# Supplementary material for: Protein Engineering of Dual-Cys Cyanobacteriochrome AM1_1186g2 for Biliverdin Incorporation and Far-Red/Blue Reversible Photoconversion
Source: Int J Mol Sci. 2019 Jun 15;20(12):2935. doi: 10.3390/ijms20122935 (PMC6628166; doi:10.3390/ijms20122935)
Supplement: Supplementary file 1 [file ijms-20-02935-s001.pdf]

Supplementary materials

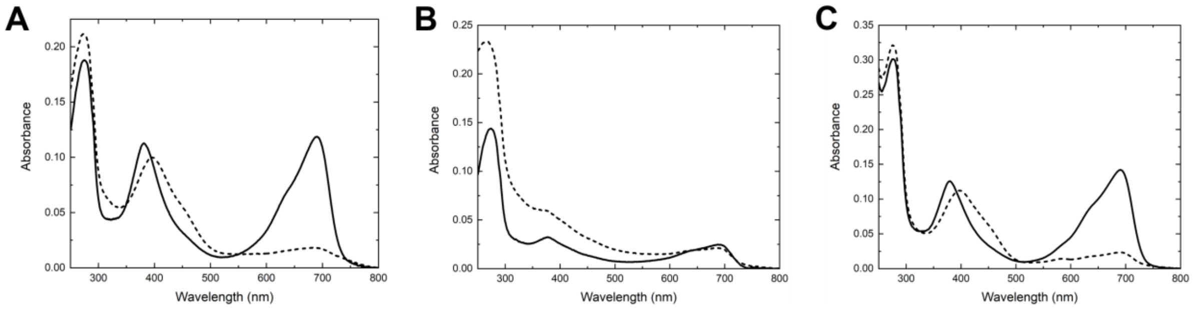

Fig. S1. Absorption spectra of KCAP_V_{2503}Q(A), KCAP_N_{2504}E (B), and KCAP_N_{2508}Q variants. The solid and dashed lines correspond to the dark states and the photoproducts responding to the far-red light, respectively.

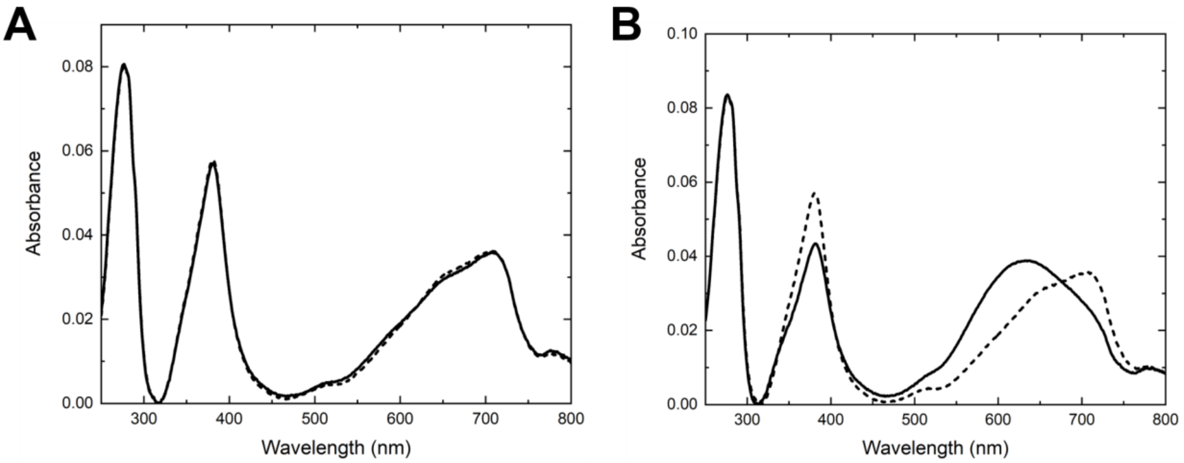

Fig. S2. Absorption spectra of acid-denatured KCAP_QV (A) Pfr form and (B) Pb form. Solid and dashed lines are spectra just after denaturation and after white light illumination, respectively.

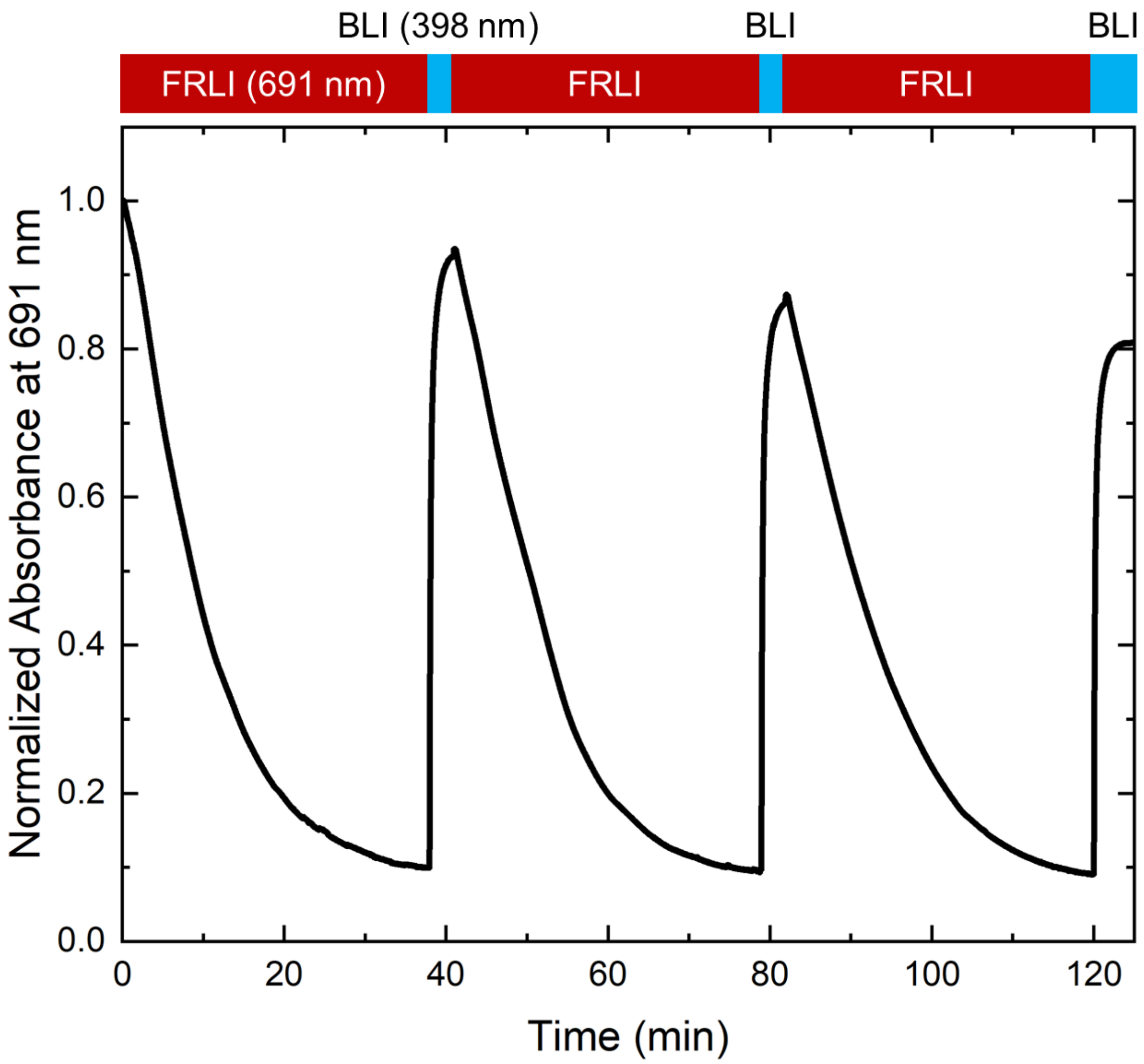

Fig. S3. Normalized absorbance changes at 691 nm during reversible photoconversion of the KCAP_QV protein. For Pfr-to-Pb or Pb-to-Pfr photoconversion, FRLI (Far-red light illumination) and BLI (Blue light illumination) cycle were repeated three times.

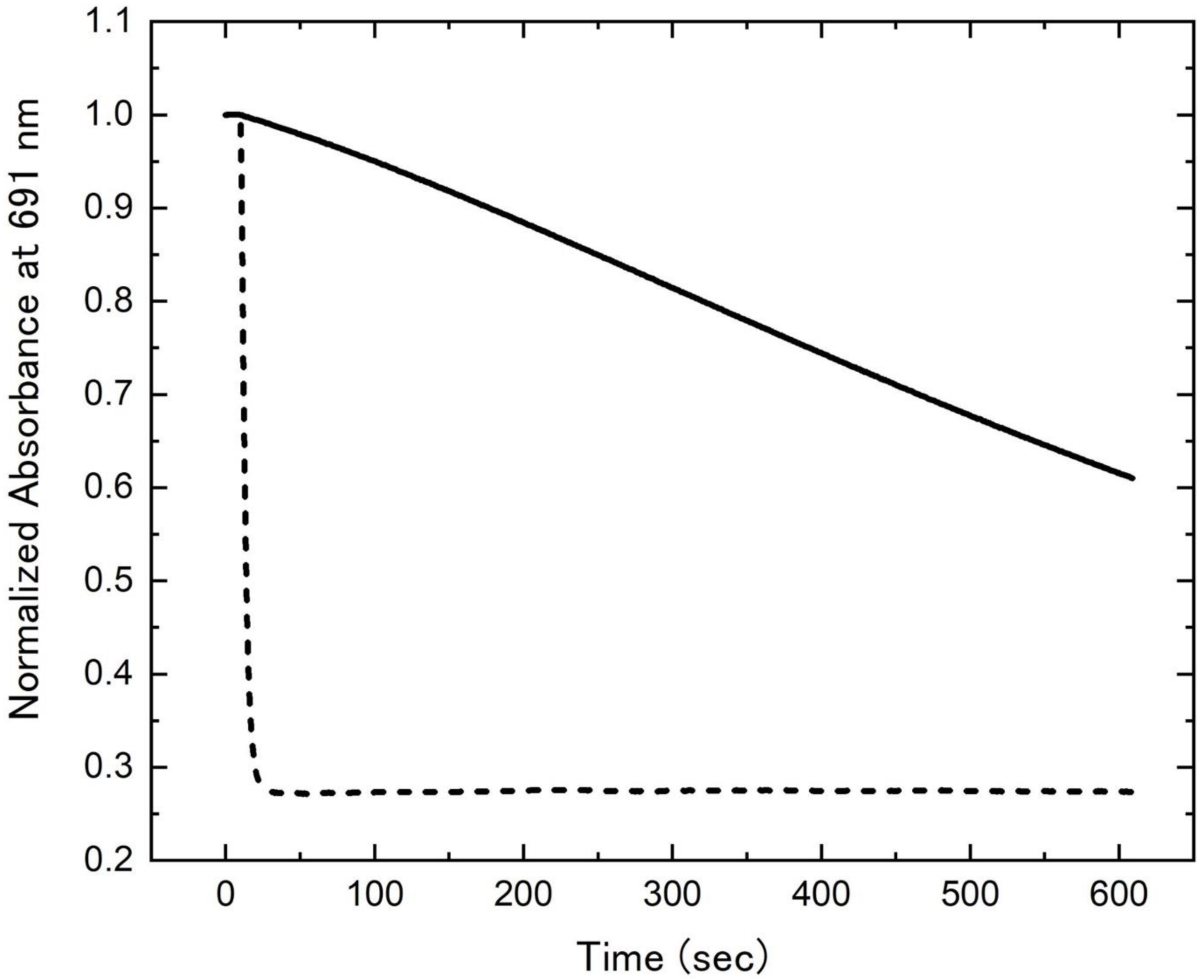

Fig. S4. Photoconversion kinetics of the KCAP_QV protein (solid line) and the AnPixJg2-BV4 protein (dashed line) from the Pfr forms. Absorption changes at 691 nm were monitored for 10 min during far-red light illumination.

Table S1. Primers used in the study

**Name**		**Sequence (5'-3')**
**RCAP**	Fw	TCGCTGCGCCCCCAACACGACTTACCAGG
	Rv	TTGGGGGCGCAGCGATTCCCTTCCTCGTT
**CAP**	Fw	GGGAATCCCTGCGCCCCCAACACGACT
	Rv	GGCGCAGGGATTCCCTTCCTCGTTGAC
**KCAP**	Fw	GGGAATAAGTGCGCCCCCAACACGACTTAC
	Rv	GGCGCACTTATTCCCTTCCTCGTTGACCAG
**KCAP_V_{2503}Q**	Fw	TTTCTGCAGAACGAGGAAGGGAATAAG
	Rv	CTCGTTCTGCAGAAAGGAGTCGTTCCA
**KCAP_N_{2504}E**	Fw	CTGGTCGAGGAGGAAGGGAATAAGTGC
	Rv	TTCCTCCTCGACCAGAAAGGAGTCGTTCC
**KCAP_N_{2508}G**	Fw	GAAGGGGGCAAGTGCGCCCCCAAC
	Rv	GAAGGGGGCAAGTGCGCCCCCAAC
**KCAP_Q_{2517}V**	Fw	ACTTACGTGGTGGATGACATTAGCGAG
	Rv	ATCCACCACGTAAGTCGTGTTGGGGGC
